# Characteristics and risk factors for severe repeat-breeder female pigs and their lifetime performance in commercial breeding herds

**DOI:** 10.1186/s40813-017-0059-0

**Published:** 2017-06-08

**Authors:** Satomi Tani, Carlos Piñeiro, Yuzo Koketsu

**Affiliations:** 1 School of Agriculture, Meiji University, Higashi-mita 1-1-1, Tama-ku, Kawasaki, Kanagawa 214-8571 Japan; 2 PigCHAMP Pro Europa S.L., c/Santa Catalina 10, 40003 Segovia, Spain

**Keywords:** Severe repeat-breeding, Lifetime performance, Herd productivity groups

## Abstract

**Background:**

Repeat-breeder females increase non-productive days (NPD) and decrease herd productivity and profitability. The objectives of the present study were 1) to define severe repeat-breeder (SRB) females in commercial breeding herds, 2) to characterize the pattern of SRB occurrences across parities, 3) to examine factors associated with SRB risk, and 4) to compare lifetime reproductive performances of SRB and non-SRB females. Data included 501,855 service records and lifetime records of 93,604 breeding-female pigs in 98 Spanish herds between 2008 and 2013. An SRB female pig was defined as either a pig that had three or more returns. The 98 herds were classified into high-, intermediate- and low-performing herds based by the upper and lower 25th percentiles of the herd mean of annualized lifetime pigs weaned per sow. Multi-level mixed-effects logistic regression models with random intercept were applied to the data.

**Results:**

Of 93,604 females, 1.2% of females became SRB pigs in their lifetime, with a mean SRB risk per service (± SEM) of 0.26 ± 0.01%. Risks factors for becoming an SRB pig were low parity, being first-served in summer, having a prolonged weaning-to-first-mating interval (WMI), and being in low-performing herds. For example, served gilts had 0.81% higher SRB risk than served sows (*P* < 0.01). Also, female pigs in a low-performing herd had 1.19% higher SRB risks than those in a high-performing herd. However, gilt age at-first-mating (*P* = 0.08), lactation length (*P* = 0.05) and number of stillborn piglets (*P* = 0.28) were not associated with becoming an SRB female. The SRB females had 14.4–16.4 fewer lifetime pigs born alive, 42.8–91.3 more lifetime NPD, and 2.1–2.2 lower parities at culling than non-SRB females (*P* < 0.05).

**Conclusions:**

We recommend that producers closely monitor the female pig groups at higher risk of becoming an SRB.

## Background

Approximately 7% of first-served female pigs are re-served in commercial herds [1]. Furthermore, a study of 539 U.S.A. herds reported that 17–20% of the re-served females had a second re-service, and also that 22–27% of the second-return females had a total of three or more re-service occurrences [2]. A recent study also indicated that culling guidelines for third-returned females are not always strictly implemented [3], and so a substantial number of severe repeat-breeder (SRB) female pigs may exist in commercial herds, thus increasing non-productive days (NPD) and affecting herd productivity [4]. Meanwhile, there is no clear and consistent definition for repeat-breeder females in previous studies. Repeat-breeding has been defined as one return [5, 6], while SRB has been defined as one to four returns and culled [7]. Furthermore, there is little information about SRB females in commercial herds, such as their lifetime performance, nor how the reproductive performance in parities before a re-service occurrence differs from that of non-SRB females.

Re-servicing has been associated with lower parity and summer serving [8–10]. Other factors that have been associated with lower farrowing rate and more returns are higher pre-service outdoor temperature and prolonged weaning-to-first-mating interval (WMI; [9, 11, 12]). Additionally, a high abortion risk, which is one of the reasons for return occurrences, is also associated with an increased number of stillborn piglets [13]. However, risk factors for SRB females have not been quantified, nor has the pattern of SRB females across parities been examined in detail. Therefore, the objectives of this study were 1) to define SRB females in commercial breeding herds, 2) characterize SRB females across parities, 3) to quantify factors associated with SRB risk, 4) to compare lifetime reproductive performances of SRB and non-SRB females, and 5) to compare reproductive performance of SRB and non-SRB sows in the parity before the SRB sows had their SRB occurrence.

## Methods

### Study herds

A consultancy firm (PigCHAMP Pro Europa S.L. Segovia, Spain) has requested all client producers to mail their data files since 1998. At the end of 2013, 98 out of the 120 Spanish client herds (82%) allowed their herd data to be used for research purposes. In 2013 the database included approximately 0.7% of all herds in Spain, one of the major pig producing regions in Europe. There are 2,568,450 female pigs in 19,630 breeding herds in Spain [14].

These herds use natural or mechanical ventilation in their farrowing, breeding and gestation barns. The lactation and gestation diets are formulated using cereals (barley, wheat and corn) and soybean meal. Also, all the herds use artificial insemination; double or triple inseminations of sows during an estrous period are practiced for breeding management. Replacement gilts in the herds were either purchased from breeding companies or were home-produced through internal multiplication programs.

### Study design, data collection and exclusion criteria

The present study was designed as a retrospective cohort study coordinating by-parity service records from herd entry to removal for female pigs entered the herds from 2008 to 2010, using the PigCHAMP recording system at the end of 2013. Service record were collected from January 2008 to June 2013, because the female pigs lived up to 3 years. At the time the records were collected, 4842 (4.8%) of the 99,533 female pigs had not yet been removed from the herds and so these records were excluded when lifetime records were analyzed. Thus, the initial data contained 517,222 service records in 465,947 parity records and lifetime records of 94,691 females in the 98 herds. Female pigs were excluded if they had NPD of more than 289 days (99th percentile; 949 records), because these data were likely to be incorrectly recorded.

Service records of sows were excluded if they met any of the following criteria: total number of pigs born was 0 pigs or 26 pigs or more (215 records; [15]); lactation length of either 0–9 days or greater than 41 days (3756 records; [16]); WMI of 36 days or more (3779 records; [16]); re-service intervals of 151 days or more (479 records). Hence, the data included 501,855 service records in 454,058 parity records of 93,604 female pigs. Additionally, records with no gilt age at first-mating (AFM) or with an AFM of either less than 160 days or more than 401 days (6767 females; [16]) were excluded when the combined gilts and sows model was used. Two datasets were created from the data, containing lifetime performance (Dataset 1) and service records for consecutive parities (Dataset 2).

### Definitions and categories

An SRB female pig was defined as a pig that had had three or more returns within the same parity, based on a modification of the definition used for RB cows [17]. The SRB risk (%) was defined as the number of SRB females divided by the number of first-served females. A re-served female pig was defined as a pig that had more than one service event within the same parity. The first, second and third returns were defined as the first, second and third returns-to-service within the same parity, respectively. A service included one or more inseminations or natural matings during an estrus period. Re-service intervals were categorized into four groups: early, regular, irregular, and late returns, with respective re-service intervals of 11 to 17, 18 to 24, 25 to 38 and 39 to 150 days post-service [8].

Annualized lifetime pigs weaned per sow was defined as the lifetime number of weaned pigs divided by the sum of the reproductive herd life days × 365 days. Reproductive herd life days was defined as the number of days from the date that the gilts was first-inseminated to its removal [18]. Lifetime NPD was defined as the number of days when a female was neither gestating nor lactating during her reproductive herd life. Lifetime pigs born alive was defined as the sum of the number of pigs born alive in a sow’s lifetime.

Three categories of herd productivity were defined on the basis of the upper and lower 25th percentiles of the herd means of annualized lifetime pigs weaned per sow: high-performing herds (> 24.7 pigs), intermediate herds (24.7 to 21.2 pigs) and low-performing herds (< 21.2 pigs). Mean (± SEM) herd size between 2008 and 2013 was 699 ± 64.3 females with a range between 81 and 3222 females.

Removal types included culling and death. Culling due to reproductive failure included culling due to being found not pregnant, repeats and abortion. Reasons for culling were recorded by producers when female pigs were culled. Served month was categorized into four seasonal groups (January to March, April to June, July to September and October to December). Three WMI groups were formed: 0 to 6 days, 7 to 12 days and 13 days or more.

### Statistical analysis

Descriptive statistics were performed using SAS version 9.3 (SAS Inst. Inc., Cary, NC). A linear mixed-effects model was applied to the two datasets using the MIXED procedure with a Tukey-Kramer multiple comparisons test. Models 1 and 2 were applied to examine the association between production factors and lifetime performances in Dataset 1 (Model 1), or previous reproductive performance by parity groups in Dataset 2 (Model 2). These models included female groups (SRB or non-SRB females), the four seasonal service groups, the three herd productivity groups and entry year as fixed effects. A random herd effect was also included in both models. A chi-square test was conducted using SAS software to compare the relative frequency (%) of re-service intervals between SRB females and non-SRB females. Also, herd size was compared between the herd groups using ANOVA.

Models 3 and 4 were used to analyze service records for gilts and sows (Model 3) and sows only (Model 4) to determine whether or not a female pig was SRB (i.e., SRB risk per service). Models 3 and 4 were also used to account for the 3-level hierarchy of individual service records within a female and for females within a herd. The models were constructed by applying multilevel generalized linear models for SRB risks with a logit link function to Dataset 2 using MLwiN version 2.29 (University of Bristol, Bristol, UK). However, initial analyses showed that the estimated variances at the sow level were very close to zero (<0.0001). Therefore, the sow level was omitted from the models for the SRB risks [19, 20]. Parameter estimation was performed using the second-order penalized quasi-likelihood method and the iterative generalized least squares algorithm in MLwiN. The models contained the four seasonal service groups, the three herd productivity groups and entry year as fixed effects. Model 3 included AFM, whereas Model 4 contained sow specific factors such as the number of stillborn piglets, the three WMI groups and lactation length. All the continuous variables were centered at their grand mean values. Non-significant variables (*P* > 0.05) were eliminated from each model. The quadratic expressions of continuous variables were examined and non-significant quadratic expressions were removed (*P* ≥ 0.05). Random herd effect was also included in the models. The adequacy of the model assumptions for the random effects was assessed by visual inspection of normal-probability plots [21].

### Intraclass correlation coefficient

The intraclass correlation coefficients (ICC) were calculated by the following equation [22] to assess the variance in SRB risk that could be explained by the herd:

ICC (records within the same herd) $={\sigma}_{\upnu}^2/\left({\sigma}_{\upnu}^2+\left({\pi}^2/3\right)\right)$, in which ${\sigma}_{\upnu}^2$ is the between-herd variance and *π* ^2^/3 is the variance at the assumed individual record level.

## Results

**Fig. 1:**
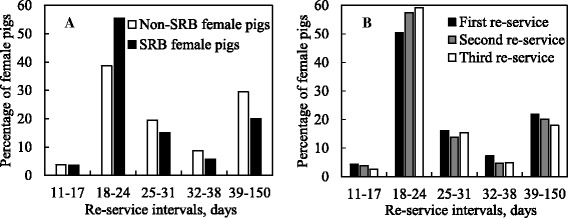
Relative frequencies (%) of re-service intervals for re-service records. **a** Re-service intervals for 5968 re-service records of severe repeat-breeder (SRB) females and 41,401 re-service records of non-SRB females. **b** Re-service intervals for 2469 first, 2008 s and 1491 third re-service records of SRB females

Mean SRB risk per service (± SEM) was 0.26 ± 0.01% (Table 1). The SRB risks were greater in low parities, with 0.57 and 0.30% SRB risks for parity 0 and 1 females, respectively, compared with only 0.07–0.21% in parity 2 or higher sows (Table 2). The SRB female pigs had more regular returns than non-SRB female pigs (*P* < 0.05; Fig. 1) but the mean (± SEM) re-service interval for SRB females was 30.8 ± 0.31 days because 20% of SRB females had late re-service intervals of 39–150 days. Also, the proportions of SRB females having regular returns at the first, second and third re-service were 50, 57 and 59%, respectively (*P* < 0.05). Third returns occurred in 92% of the 98 herds. Larger herd size was associated with herd productivity groups; low-performing herds (Mean ± SEM: 482 ± 60 females) and intermediate herds (617 ± 83 females) had smaller herd sizes than high-performing herds (1095 ± 169 pigs; *P* < 0.01).

**Table 1:** Reproductive data for female pigs in 98 commercial herds from 2008 to 2013

			Range	
Measurements	N	Mean ± SEM	Minimum	Maximum
Lifetime records				
Parity at removal	93,604	4.6 ± 0.01	0	13
Gilt age at first-mating, days old^a^	86,837	251.6 ± 0.15	160	400
Number of lifetime pigs born alive	86,749	58.7 ± 0.11	0	202
Lifetime non-productive days of female pigs	93,604	84.9 ± 0.16	0	289
Female life days	88,972	942.8 ± 1.26	28	1703
SRB females, %	93,604	1.2 ± 0.03	–	–
Parity records				
Parity at service	454,058	2.6 ± 0.01	0	12
Records with two returns or more, %	454,058	1.37 ± 0.01	–	–
SRB risk for first served females, %	454,058	0.26 ± 0.01	–	–
Number of pigs born alive	360,454	12.1 ± 0.01	0	25
Number of stillborn piglets	360,454	0.9 ± 0.01	0	19
Lactation length, days	360,454	23.4 ± 0.01	10	41
Weaning-to-first-mating interval, days	360,454	5.8 ± 0.01	0	35
Service records				
Number of services	501,855	1.1 ± 0.01	1	9
Re-service intervals for RB females, days	4258	30.8 ± 0.31	11	145

*SRB* severe repeat-breeder

^a^The remaining records (93,604-N) were regarded as missing records

**Table 2:** Severe repeat-breeder (SRB) risks in served female pigs by parity group

	Parity						
Measurements	0	1	2	3	4	5	6 or later
Number of serviced records	93,604	78,243	70,596	62,258	53,371	43,634	52,352
Number of first return records	11,458	8734	5625	4730	3772	2694	2614
First return occurrences, %	12.2	11.2	8.0	7.6	7.1	6.2	5.0
Number of second return records	2325	1324	834	695	490	297	290
Number of culled records after the second return^a^	246	142	87	105	68	55	49
Number of removed records after the second return^b^	394	232	168	136	90	68	64
Number of SRB females^c^	532	235	145	104	70	34	38
SRB risk for first-served females, %	0.57	0.30	0.21	0.17	0.13	0.08	0.07

^a^Number of females culled due to reproductive failure

^b^Other removed records included females removed due to non-reproductive problems (e.g., lameness, diseases or death)

^c^The 8 pigs (1158-1150) had twice SRB in their lifetime

With regard to lifetime performance, 1150 (1.2%) of the 93,604 females became SRB pigs in their lifetime (Table 3). The SRB females had 3.0–4.3 fewer annualized lifetime pigs weaned, 14.4–16.4 fewer lifetime pigs born alive, 42.8–91.3 more lifetime NPD and 2.1–2.2 lower parities at culling than non-SRB females (*P* < 0.01; Table 3). Additionally, females that became SRB in parity 1 had 5.2% lower farrowing rates in their previous parity than non-SRB females (*P* < 0.01; Table 4). However, there was no difference between SRB and non-SRB female groups for WMI (*P* ≥ 0.07).

**Table 3:** Comparisons of lifetime performance between three female return-to-service groups

	Female return groups					
	Non-return		Return once or twice, but not SRB		SRB	
Lifetime performance measurements	N	Mean (± SE)	N	Mean (± SE)	N	Mean (± SE)
Annualized lifetime number of pigs born alive	56,803	27.6 (0.15)^a^	29,093	25.7 (0.15)^b^	853	21.4 (0.26)^c^
Annualized lifetime number of pigs weaned	56,803	23.6 (0.10)^a^	29,093	22.3 (0.10)^b^	853	19.3 (0.20)^c^
Lifetime non-productive days	61,348	74.0 (1.26)^c^	31,106	122.5 (1.26)^b^	1150	165.3 (1.74)^a^
Lifetime pigs born alive	56,803	58.9 (0.80)^b^	29,093	60.9 (0.80)^a^	853	44.5 (1.35)^c^
Gilt age at first-mating, days old	57,022	254.6 (3.18)^ab^	28,771	253.7 (3.18)^b^	1044	254.7 (3.33)^a^
Number of parity at removal	61,348	4.7 (0.07)^b^	31,106	4.9 (0.07)^a^	1150	2.9 (0.10)^c^
Number of parity at culling	51,496	4.9 (0.08)^b^	27,389	5.0 (0.08)^a^	1052	2.8 (0.11)^c^
Number of parity at death	9852	3.2 (0.07)^c^	3717	4.1 (0.07)^a^	98	3.5 (0.24)^b^
Female life days	58,482	950.2 (10.28)^b^	29,407	1029.5 (10.28)^a^	1083	807.7 (14.99)^c^
Total cumulative re-service intervals, days	61,348	0.0 (0.00)	31,106	56.0 (1.05)^b^	1150	111.3 (1.52)^a^

*SE* standard error, *SRB* severe repeat-breeder

^a-c^Mean values within a row followed by different letters differ (*P* < 0.05)

**Table 4:** Comparisons of reproductive performances^1^ between severe repeat-breeder (SRB) and non-SRB sows

	Female pigs	
	Non-SRB	SRB
Parity	Mean (± SE)	Mean (± SE)
Farrowing rate for first service in the previous parity, %		
Parity 1	87.6 (0.54)^a^	82.4 (2.07)^b^
Parity 2	88.6 (0.49)	84.6 (2.51)
Parity 3	92.4 (0.38)	96.5 (2.45)
Parity 4	92.9 (0.34)	89.5 (2.88)
Parity 5	93.2 (0.36)	93.2 (3.96)
Parity 6	94.5 (0.31)	93.4 (4.24)
Weaning-to-first-mating interval in the previous parity, days		
Parity 2	8.3 (0.19)	7.5 (0.73)
Parity 3	6.4 (0.10)	6.8 (0.59)
Parity 4	6.1 (0.10)	6.3 (0.67)
Parity 5	5.9 (0.10)	5.2 (0.88)
Parity 6	5.7 (0.09)	5.6 (0.98)

^1^Reproductive performances are measured at the previous parity before the SRB females became SRB

^a,b^Mean values within a row fallowed by different letters differ (*P* < 0.05)

In both the first-served female (sows and gilts) model and the sows only model, an SRB risk was associated with low parity (i.e., parities 0 and 1), being first-served in summer and being fed in low-performing herds (*P* < 0.01; Table 5). Also, in the sows only model an SRB risk was associated with sows having prolonged WMI. For example, served gilts had 0.43 to 0.81% higher SRB risk than served sows (*P* < 0.01; Table 6). Additionally, females in a low-performing herd had 0.73 to 1.19% higher SRB risks than those in a high-performing herd. Also, the SRB risk of sows that had a WMI of 7–12 days was 0.10% higher than that of sows which had a shorter WMI of only 0–6 days (*P* < 0.05; Table 6). However, AFM (*P* = 0.08), lactation length (*P* = 0.05) and fewer stillborn piglets (*P* = 0.28) were not associated with being an SRB female. With regard to the ICC, the random herd effect explained 25.5% and 30.1% of total variance values for SRB risk in the female (gilts and sows) model and the sow only model, respectively (Table 5).

**Table 5:** Estimates of factors in the final logistic regression models of the severe repeat-breeder risks

	Gilts and sows		Sows only	
Fixed effects and variance	Estimate (± SE)	*P*-value	Estimate (± SE)	*P*-value
Intercept^a^	− 8.584 (0.283)	<0.01	− 8.836 (0.325)	<0.01
Parity groups		<0.01		<0.01
Parity 0	2.170 (0.149)		–	
Parity 1	1.534 (0.155)		1.562 (0.155)	
Parity 2–5	0.827 (0.151)		0.862 (0.151)	
First-served month group		<0.01		<0.01
Apr.-Jun.	0.028 (0.075)		0.009 (0.101)	
Jul.-Sep.	0.302 (0.071)		0.338 (0.094)	
Oct.-Dec.	− 0.015 (0.076)		− 0.003 (0.102)	
Herd productivity groups		<0.01		<0.01
Intermediate-performing herds	1.636 (0.288)		1.856 (0.339)	
Low-performing herds	2.473 (0.324)		2.764 (0.377)	
WMI groups				0.03
WMI 7–12 days	–		0.280 (0.111)	
WMI 13 days or more	–		− 0.063 (0.134)	
Herd variance	1.12 (0.18)		1.42 (0.24)	
ICC (records within the same herd), %	25.5		30.1	

*SE* standard error, *ICC* intraclass correlation coefficient, *WMI* weaning-to-first-mating interval

**Table 6:** Comparisons between factors for severe repeat-breeder (SRB) risks per service

	Gilts and sows	
Explanatory variables	N	SRB risk (99% CI)
Parity groups		
0	93,604	0.92 (0.67–1.26)^a^
1	78,243	0.49 (0.35–0.68)^b^
2–5	229,859	0.24 (0.17–0.34)^c^
6 or more	52,352	0.11 (0.07–0.18)^d^
First-served month group		
Jan.-Mar.	113,178	0.29 (0.21–0.42)^b^
Apr.-Jun.	114,322	0.30 (0.21–0.44)^b^
Jul.-Sep.	112,615	0.40 (0.27–0.56)^a^
Oct.-Dec.	113,943	0.29 (0.20–0.41)^b^
Herd productivity groups		
Low-performing herds	65,565	1.30 (0.73–2.22)^a^
Intermediate-performing herds	196,848	0.57 (0.37–0.85)^b^
High-performing herds	191,645	0.11 (0.06–0.21)^c^
WMI groups^1^		
WMI 0–6 days	315,071	0.26 (0.19–0.39)^b^
WMI 7–12 days	22,633	0.36 (0.23–0.57)^a^
WMI 13 days or more	22,750	0.27 (0.15–0.42)^ab^

*CI* confidence interval, *WMI* weaning-to-first-mating interval

^a-c^Mean values within a group with different letters are different (*P* < 0.05)

^1^The WMI groups were compared in the model containing sows only (*N* = 360,454)

## Discussion

Our study showed that one fifth of the 1.2% of first-served females that became RB pigs had re-service intervals of 39–150 days which would greatly increase NPD. Also, re-serving is one of the risk factors for abortion [13] and further increasing NPD. Re-service intervals and culling intervals account for 70% of NPD [4] which must be minimized to improve sow productivity. Additionally, the fact that SRB females had more regular re-service intervals (18–24 days) than non-SRB females indicates that SRB females are more likely to have either conception failure or failure of maternal recognition [8]. Other factors may also have contributed to the greater likelihood of SRB females having regular re-service, such as semen quality and storage [23], as well as timing of insemination [24].

In our study we found clear parity effects on the likelihood of females becoming SRB, with a higher risk in low parity females. One possible reason for the higher SRB risks in low parity females is that culling pressure for low parity females is not as high as that for aged sows, because gilts and parity 1 sows are still growing animals and producers want to recover the initial cost of a replacement gilt [5, 25]. Also, some sows with a reproductive problem may be culled before parity 2. Furthermore, recent data from Spanish, Portuguese and Italian herds showed that 41 and 36% of respective first-returned gilts and parity 1 sows had a return recurrence in the same or later parity [20]. Therefore, it is critical to have more accurate estrus detection and strict selection criteria for gilts in order to decrease both the number of SRB gilts and parity 1 sows and NPD in breeding herds. However, in our study low parity SRB females had shorter estrus duration than equivalent non-SRB females (Fig. 1), and it has also been reported that low parity females have subtle estrus behavior that is more difficult to detect [26]. Therefore, these differences in estrus characteristics make it difficult to identify the correct insemination timing in SRB females. One way to improve gilt development would be to include a boar stimulation protocol [27], which would help to distinguish between gilts that would become fertile females in breeding herds and those that are more likely to become SRB females.

In our study, females that were served in summer had a 0.1% higher SRB risk than those served in winter. Therefore, it appears that high temperature or low lactational feed intake in summer can increase conception failure or pregnancy loss in served females. Other research has also shown that parity 1 sows are more sensitive than parity 2 or higher sows to high temperature for increasing returns to re-service [8]. It has been suggested that a pregnancy loss in summer is related to a combination of low GnRH secretion, decreased LH release impaired ovarian follicle development and consequently degraded corpora lutea functions that produce low progesterone concentrations [28].

The WMI effect on sows becoming SRB could be related to low gonadotropin secretion and possibly impaired endocrine systems. For example, prolonged WMI in sows is thought to be due to low LH secretion caused by low feed intake during lactation [29]. Therefore, in this study we found that the main risk factors for SRB were low parity (0 or 1), being first-served in summer and having WMI 7–12 days. These findings are consistent with previous studies in the U.S.A., Sweden and Japan reporting a higher re-service risk associated with the same three factors [8, 10, 30].

We also found that herd productivity affected SRB risk. This result suggests that heat detection, pregnancy diagnosis, as well as culling policy and implementation were not good enough in the low-performing herds, probably because small-sized low-performing herds do not have sufficient resources for advanced production systems, sufficient gilt pool, equipment or professional workers. Also, herds with more SRB females will have decreased reproductive productivity and so would become low-performing herds. In contrast, large-sized high-performing herds are likely to be able to employ more skilled workers and have better facilities than small-to mid-sized low-performing herds [31]. Finally, the lack of any association in our study between SRB risks and either lactation length, AFM or the number of stillborn piglets indicates that SRB risks were not greatly influenced by these factors in the studied herds.

Regarding lifetime reproductive performance, the fact that SRB females had 90 more NPD than non-SRB females suggests that producers’ culling guidelines were not strictly implemented for returned females in the studied herds. Keeping SRB females too long in a herd increases NPD and decreases efficiency of the sows, because the SRB females produce pigs 30% (21.4/ 27.6 annualized lifetime pigs born alive) less efficiently than non-SRB females, and their mean re-service interval of 55 days increase NPD.

The present study shows that low parity SRB sows had a lower farrowing rate in their previous parity than non-SRB sows in the same parity. It is likely that some of the low parity SRB sows had a latent reproductive problem that caused them to have a lower farrowing rate in the earlier parity, even though it had not yet become serious enough for them to require a re-service. One possible reproductive problem in low parity sows is ovary cysts, and a recent study reported that sows with ovary cysts had 20% more returns to estrus than sows without ovary cysts [32]. In contrast, the lack of any difference between parity 1 or higher SRB and non-SRB sows for farrowing rate and WMI sows indicates that the reason that the sows in parity 1 or higher became SRB was due to problems such as lactation and post weaning period. Finally, in the present study, the relatively high ICC for herd variance of 25.5% for the gilts and sows model and 30.1% for the sows only model indicates that there was a substantial effect of the herds on SRB, for example due to differences in heat detection programs or culling policy.

There are some limitations that should be noted when interpreting the results of the present study. This was an observational study performed using commercial herd data. Health status, nutritional programs, genotype, semen quality, proportions of double and triple inseminations, cysts and early or late abortion were not considered in the analyses. However, even with such limitations, this research provides valuable information for pig producers and veterinarians about SRB females in commercial herds, and the relationships between the risk of having SRB females and production factors.

Finally, our definition of SRB pigs is different from previous reports about repeat-breeding which sometimes is defined as one return [5, 6] and sometimes as one to four returns [7]. In our study, the objective was to examine severe repeat-breeder females, so we defined SRB females as having three or more returns in the same parity.

## Conclusions

We recommend that producers closely monitor high risk female groups to reduce their returns-to-service. The high risk groups include mated gilts and parity 1 sows, females being served in summer, and females having prolonged WMI, especially those in low performing herds.

## Abbreviations

NPD: Non-productive days
AFM: Gilt age at first-mating
ICC: Intraclass correlation coefficients
SRB: Severe repeat-breeder
WMI: Weaning-to-first-mating interval
